# Measuring the Performance of Neural Models

**DOI:** 10.3389/fncom.2016.00010

**Published:** 2016-02-10

**Authors:** Oliver Schoppe, Nicol S. Harper, Ben D. B. Willmore, Andrew J. King, Jan W. H. Schnupp

**Affiliations:** ^1^Department of Physiology, Anatomy, and Genetics, University of OxfordOxford, UK; ^2^Bio-Inspired Information Processing, Technische Universität MünchenGarching, Germany

**Keywords:** sensory neuron, receptive field, signal power, model selection, statistical modeling, neural coding

## Abstract

Good metrics of the performance of a statistical or computational model are essential for model comparison and selection. Here, we address the design of performance metrics for models that aim to predict neural responses to sensory inputs. This is particularly difficult because the responses of sensory neurons are inherently variable, even in response to repeated presentations of identical stimuli. In this situation, standard metrics (such as the correlation coefficient) fail because they do not distinguish between explainable variance (the part of the neural response that is systematically dependent on the stimulus) and response variability (the part of the neural response that is not systematically dependent on the stimulus, and cannot be explained by modeling the stimulus-response relationship). As a result, models which perfectly describe the systematic stimulus-response relationship may appear to perform poorly. Two metrics have previously been proposed which account for this inherent variability: Signal Power Explained (*SPE*, Sahani and Linden, 2003), and the normalized correlation coefficient (*CC*_*norm*_, Hsu et al., 2004). Here, we analyze these metrics, and show that they are intimately related. However, *SPE* has no lower bound, and we show that, even for good models, *SPE* can yield negative values that are difficult to interpret. *CC*_*norm*_ is better behaved in that it is effectively bounded between −1 and 1, and values below zero are very rare in practice and easy to interpret. However, it was hitherto not possible to calculate *CC*_*norm*_ directly; instead, it was estimated using imprecise and laborious resampling techniques. Here, we identify a new approach that can calculate *CC*_*norm*_ quickly and accurately. As a result, we argue that it is now a better choice of metric than *SPE* to accurately evaluate the performance of neural models.

## 1. Introduction

Evaluating the performance of quantitative models of neural information processing is an essential part of their development. Appropriate metrics enable us to compare different models and select those which best describe the data. Here, we are interested in developing improved metrics to assess models of the stimulus-response relationships of sensory neurons, in the challenging (but common) situation where the stimulus-response relationship is complex, and neuronal responses are highly variable. In this case, the development of appropriate performance metrics is not trivial, and so there is a lack of consensus about which metrics are to be used.

The classical way to record and model neural responses has been to repeatedly present an animal with a small, well-defined set of stimuli (such as sinusoidal gratings of different orientations, or sounds of different frequencies). The neural responses to repeated presentations of each stimulus are then averaged. Using a small stimulus set, it may be possible to present the same stimulus enough times that this averaging succeeds in reducing the effect of neuronal response variability (Döerrscheidt, 1981). It may then be possible to produce models which accurately describe the relationship between the stimulus and the averaged responses. These models can then be accurately evaluated by comparing the modeled and actual neuronal responses using standard metrics such as correlation coefficient. Under these circumstances, the correlation coefficient may be appropriate and can easily be interpreted—a poor model will have a correlation coefficient close to 0, a perfect model will have a correlation coefficient close to 1, and the square of the value of the correlation coefficient equals the proportion of the variance in the neural responses that the model is able to account for.

However, recent work in sensory neuroscience has increasingly focused on the responses of neurons to complex stimuli (Atencio and Schreiner, 2013; David and Shamma, 2013), and even natural stimuli (Prenger et al., 2004; Asari and Zador, 2009; Laudanski et al., 2012). For such stimuli, even very sparse sampling of the stimulus space may require the presentation of very large numbers of different stimuli (at least of order 2^*d*^ for *d* stimulus dimensions; also see Shimazaki and Shinomoto, 2007). This makes it difficult to repeatedly present stimuli enough times for response variability to simply average out. Estimating mean responses for a particular stimulus is thus subject to sampling noise, and in addition to that, the neuron under study may also be “intrinsically noisy” in the sense that only a small proportion of the response variability may be attributable to variability of the stimulus. Such situations are very common in sensory neuroscience, and they can render the use of correlation coefficients to evaluate the performance of models that map stimuli to responses very misleading. If only a fraction of the neural response variability is stimulus linked, then even a perfect model of that stimulus linkage will only ever be able to account for some fraction of the variance in the observed neural response data. This places a limit on the maximum correlation coefficient that can be achieved, and the interpretation of the raw correlation coefficients becomes ambiguous: for example, a relatively low correlation coefficient of 0.5 might be due to an excellent model of a noisy dataset, or of a rather poor model of a dataset with very low intrinsic and sampling noise, or something in between.

Different approaches for taking neural variability into account when measuring model performance have been developed. To get an unbiased estimate of *mutual information*, Panzeri and Treves (1996) suggested a method to extrapolate information content to an infinite number of trials (also see Atencio et al., 2012). Roddey et al. (2000) compared the coherence of pairs of neural responses to independent stimulus repetitions to derive a *minimum mean square error (MMSE)* estimator for an optimal model. The difference between the model prediction error and the MMSE of an optimal model allows the quantification of the model performance relative to the best possible performance given the neural variability.

Based not only on pairs, but even larger sets of neural responses to independent stimulus repetitions, Sahani and Linden developed the very insightful decomposition of the recorded signal into *signal power* and *noise power* (Sahani and Linden, 2003). This has lead to the *signal power explained (SPE)*, a measure based on *variance explained* which discounts “unexplainable” neural variability. While the work of Roddey et al. (2000) was already based on the differentiation between explainable and unexplainable neural response components, Sahani and Linden (2003) provided explicit estimations for those components. The *SPE* measure has been widely adopted, albeit under various names such as *predictive power, predicted response power, and relative prediction success* (Sahani and Linden, 2003; Machens et al., 2004; Ahrens et al., 2008; Asari and Zador, 2009; Rabinowitz et al., 2012). Also, it has been used as a basis for specific variants of measures for model performance (Haefner and Cumming, 2009).

Focusing on coherence and the correlation coefficient, Hsu and colleagues developed a method to normalize those measures by their upper bound (*CC*_*max*_), which is given by the inter-trial variability (Hsu et al., 2004). This yields the *normalized correlation coefficient* (*CC*_*norm*_). Following their suggestion, the upper bound can be approximated by looking at the similarity between one half of the trials and the other half of the trials (*CC*_*half*_). This measure has also been used by Gill et al. (2006) and Touryan et al. (2005). Others used the absolute correlation coefficient and controlled for inter-trial variability by comparing the absolute values with *CC*_*half*_ (Laudanski et al., 2012).

The two metrics *SPE* and *CC*_*norm*_ have been developed independently, but they both attempt—in different ways—to provide a method for assessing model performance independent of neuronal response variability. Here, we here analyze these metrics, show for the first time that they are closely related, and discuss the shortcomings of each. We provide a new, efficient way to directly calculate *CC*_*norm*_ and show how it can be used to accurately assess model performance, overcoming previous shortcomings.

## 2. Criteria of model evaluation

Neural responses are often measured as the membrane potential (Machens et al., 2004; Asari and Zador, 2009) or as the time-varying firing rate (Sahani and Linden, 2003; Gill et al., 2006; Ahrens et al., 2008; Rabinowitz et al., 2011; Laudanski et al., 2012; Rabinowitz et al., 2012) (which we will use without loss of generality). Thus, a measure of performance for such models should quantify the similarity of the predicted firing rate *ŷ* and the recorded firing rate *y* (also known as the peri-stimulus time histogram, PSTH):

(1) $$\begin{array}{l} y\left(t\right)=\frac{1}{N}\overset{N}{\underset{n=1}{\sum }}R_{n}\left(t\right) \end{array}$$

where *R*_*n*_ is the recorded response of the *n*th stimulus presentation and *N* is the total number of stimulus presentations (trials). Both *R*_*n*_(*t*) and *y*(*t*) are a function of the time bin *t*, but the argument *t* will not be shown for rest of the manuscript. Each value of the vector *R*_*n*_ contains the number of spikes that were recorded in the corresponding time bin. Note that, given the trial-to-trial variability of sensory responses, the recorded firing rate *y* is only an approximation of the true (but unknown) underlying firing rate function that is evoked by the presentation of a stimulus (also see Kass et al., 2003). It is a sample mean which one would expect to asymptote to the true mean as the number of trials increases (*N* → ∞). As will be discussed in detail at a later point, the difference between the recorded firing rate *y* and the true underlying firing rate is considered to be noise under the assumption of rate coding. This is the unexplainable variance that reflects the variability of the neuronal response. As the number of trials increases, the difference between *y* and the true underlying firing rate decreases and so does the non-deterministic and thus unexplainable variance in the signal.

With the recorded firing rate *y* being the target variable for the prediction *ŷ*, a measure of model performance needs to quantify the similarity between both signals, i.e., the prediction accuracy. Note that model performance is not necessarily the same as prediction accuracy (see next section).

## 3. Signal power explained

Two somewhat related metrics which are widely applied in statistics are the “coefficient of determination” (*CD*) and the “proportion of variance explained” (*VE*). Both these metrics essentially incorporate the assumption that the quantitative observations under study—in our case the responses of a sensory neuron or neural system—are the sum of an essentially deterministic process which maps sensory stimulus parameters onto neural excitation, plus an additive, stochastic noise process which is independent of the recent stimulus history (Sahani and Linden, 2003). Consequently, if a model is highly successful at predicting the deterministic part, subtracting the predictions from the observations should leave only the noise part, but if its predictions are poor, the residuals left after subtracting predictions from observations will contain both noise and prediction error. Thus, smaller residuals are taken as a sign of better prediction. The *CD* is an index that quantifies the size of the residuals relative to the size of the original observation in a quite direct manner as a sum of squares, and subtracts that unaccounted for proportion from 100% to give an estimate of the proportion of the signal that is accounted for by the model. Thus

(2) $$\begin{array}{l} CD=1-\frac{\underset{t}{\sum }{\left(y\left(t\right)-ŷ\left(t\right)\right)}^{2}}{\underset{t}{\sum }y{\left(t\right)}^{2}} \end{array}$$

The *VE* quantifies prediction accuracy in a largely analogous manner, but instead of using the “raw” sum of squares of the observations and the residuals, it instead uses the respective sample variances, measured around their respective sample means:

(3) $$\begin{array}{ll} VE=1-\frac{Var\left(y-ŷ\right)}{Var\left(y\right)} & \; \end{array}$$

This makes the *VE* insensitive to whether the mean of the predicted responses closely corresponds to the mean of the observed responses over all *t*, which can sometimes be an advantage. Even small errors (biases) in the mean of the prediction can be penalized quite heavily by the *CD* measure as these will accumulate over every sample. The *VE* measure can be thought of as deeming such biases as unimportant, and focusing solely on how well the model predicts the trends in the responses as a function of *t*.

*CD* and *VE* have a long established history in statistics, but neither provide an unambiguous measure of model performance because large amounts of residual variance, and therefore low *VE* or *CD* values, could arise either if the model provides a poor approximation to underlying deterministic and predictable aspects of the process under study, or if the model captures the deterministic part of the process perfectly well, but large amounts of fundamentally unpredictable noise in the system nevertheless cause the amount of residual variance to be large. In other words, even a perfect model cannot make perfect predictions, because the neuronal response has a non-deterministic component. Even if the model was completely identical to the neuron in every aspect, it would nevertheless be unable to explain 100% of the variance in the neuronal responses because the PSTHs collected over two separate sets of stimulus presentations cannot be expected to be identical and the first set does not perfectly predict the second. Furthermore, since the number of trials *N* used to determine any one PSTH is often rather low for practical reasons, observed PSTHs are often somewhat rough, noisy estimators of the underlying neural response function (also see Döerrscheidt, 1981; Kass et al., 2003; Shimazaki and Shinomoto, 2007). A good measure of model performance for sensory neural systems should take these considerations into account and judge model performance relative to achievable, rather than total, prediction accuracy. Such considerations led Sahani and Linden (2003) to introduce metrics which split the variance in an observed PSTH, the *total power* (*TP*), into the *signal power* (*SP*), which depends deterministically on recent stimulus history, and the stochastic *noise power* (*NP*). Only the *SP* is explainable in principle by a model, and the *signal power explained* (*SPE*) thus aims to quantify model performance relative to the best achievable performance. *SPE* is defined as:

(4) $$\begin{array}{rcl} SPE & = & \frac{Var\left(y\right)-Var\left(y-ŷ\right)}{SP} \end{array}$$

(5) $$\begin{array}{rcl} SP & = & \frac{1}{N-1}\left(N\times Var\left(y\right)-TP\right)\\ TP & = & \left(N-1\right)\times \overset{N}{\underset{n=1}{\sum }}Var\left(R_{n}\right) \end{array}$$

*SPE* is quantified as the ratio of the *explained* signal power relative to the *explainable* signal power^1^. The *explained* signal power is calculated by subtracting the variance of the residual (the error) from the total variance in the observed firing rate. The *explainable* signal power *SP* is calculated according to formulas developed in Sahani and Linden (2003) and reproduced below (Equation 13). Good models will yield small error variance and thus a large *SPE* - and vice versa. However, this measure lacks an important characteristic: it is not bounded. While a perfect model would yield an SPE of 100%, the measure has no lower bound and can go deeply into negative values when the variance of the error is bigger than the variance of the neural signal. This shortcoming of the *SPE* metric can be exposed by reformulating parts of the equation. First, observe that for two random variables *X* and *Y* the variance of their difference can be expressed as :

(6) Var(Y−X)=Var(Y)+Var(X)−2×Cov(X,Y)

Applying this reformulation to Equation 5 reveals that:

(7) $$SPE=\frac{Var(y)-Var(y-\hat{y})}{SP}=\frac{2\times Cov(y,\hat{y})-Var(\hat{y})}{SP}$$

Consider a particularly bad model, which produces predictions that are no better than the output of a random number generator. The covariance between the predictions and the neural responses will then be close to zero, but the variance (i.e., the power of the predicted signal) of the predicted signal may nevertheless be large. The *SPE* for such a model would be a negative number equal to −*Var*(*ŷ*)/*SP*. This is a counterintuitive property of the *SPE* metric: the “proportion of the signal power that is explained” by a list of random numbers should be zero, not negative. Also, two bad models that are equally unable to capture the trends of the signal they are trying to predict and thus have near zero covariance may nevertheless have widely different negative *SPE* values, but how negative their *SPE* values are may have little systematic relationship to how large their prediction errors are on average, which makes small or negative *SPE* values very difficult to interpret.

**Figure 1:**
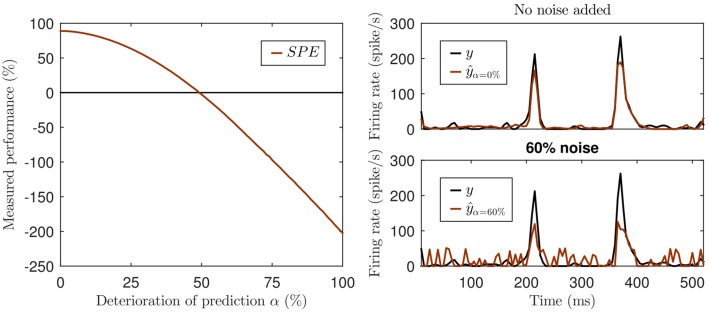
**Illustration of the missing lower bound of SPE**. Left panel: The simulation was created by adding increasing white noise (*w*) to an actual prediction *ŷ* generated by an artificial neural network: *ŷ*_α_ = α*w* + (1 − α)*ŷ* with 0% ≤ α ≤ 100%, negative values of the deteriorated *ŷ*_α_ set to 0. Top right panel: The original prediction *ŷ* of the neural network (red) and the actual neural response (black). Lower right panel: The deteriorated prediction at a noise level of 60% (*SPE* = −39%).

This can be illustrated with a simple hypothetical example. Imagine a visual cortex simple cell responding to a sinusoidal contrast grating stimulus with a sinusoidal modulation of its firing rate, so its observed response is a sine wave, let's say, of an amplitude of ±1 spikes/s around a mean firing rate of 10 spikes/s at a modulation rate of 1 Hz. Let us further assume that model A predicts sinusoidal firing at a 2 Hz modulation rate with an amplitude of ±2 spikes/s around a mean of 10 spikes/s, and model B predicts a sinusoidal firing at 2 Hz with an amplitude of ±1 spikes/s around a mean of 100 spikes/s. Since neither model A nor B correctly predicted the rate of the sinusoidal firing rate modulations, and because sine waves of different frequencies are orthogonal, both models will have covariance of zero with the observed data. Thus, they have a negative *SPE*, as the signal variance is greater than zero. And because model A predicted larger amplitude fluctuations than model B, and thus has greater variance, the *SPE* of model A will be more negative than that of model B, which one might be tempted to interpret to mean that model A performed worse. However, the discrepancy or prediction error between observed and predicted rates for model A will never be more than 3 spikes/s, while that of model B will never be less than 88 spikes/s, and the more negative *SPE* of model A contrasts sharply with the fact that model A produces a much smaller mean squared prediction error than model B. Furthermore *SPE* can yield negative values even when there is a reasonable amount of covariance between model and prediction, if the variance in the predicted signal is also sizable. This is illustrated in Figure 1. Not only is such a measure rather hard to interpret, but it can be misleading. Due to the missing lower bound the values can not only become negative, but the exact value also depends on the variance of the prediction. Consider the prediction with 60% noise in the lower right panel of Figure 1. While this prediction is surely not a good one, the fact that data and model prediction co-vary to a fair degree is nevertheless readily apparent, and it would be hard to argue that a model predicting a flat, arbitrary, constant firing rate (say 800 spikes/s) would be a better alternative. Yet the variance of any predicted constant firing rate would be zero and so would be their *SPE*, which may seem indicative of a “better explanatory power” of the constant rate model compared to the “60% noise” added model of Figure 1 with its *SPE* = −39%, but the noisy model clearly captures some of the major peaks in the data while constant rate models don't even try.

These examples illustrate that models can be thought of as being wrong in different ways. They can be “biased,” predicting an incorrect overall mean response rate, they can be “scaled wrong,” predicting fluctuations that are too small or too large, or they can fail to predict the trends and dynamics of the data, leading to near zero covariance between observation and prediction. Different metrics of model performance will differ in how sensitive they are to these different types of error. *SPE* is sensitive both to poor scaling and poor covariance, but not to bias. Some might argue, quite reasonably, that this combined sensitivity to two types of error is a virtue: When *SPE* values are large then we can be confident that the model achieves both good covariance and good scaling. However, the downside of this joint sensitivity is that small or negative *SPE* values have limited diagnostic value because they could be due to small covariance or to overestimated (but not underestimated) predicted variance, or some combination of the two. Consequently, as we will illustrate further in section 6, *SPE* values below about 0.4 become very difficult to interpret, and may be much at odds with other commonly used measures of model performance.

Negative values of the *SPE* have been previously reported (Machens et al., 2004; Ahrens et al., 2008) and have been interpreted as a sign of overfitting of the model. Overfitting usually manifests itself as a decline in covariance between data and predictions in cross-validation tests, and as such would result in small or negative *SPEs*, but because *SPE* will become negative for any prediction which has a residual variance that is larger than the variance of the target signal, negative *SPE* is not a specific diagnostic of overfitting. Also negative *SPEs* do not necessarily imply that a model performs worse than a “null model” which predicts constant responses equal to the mean firing rate. In fact, any model predicting any arbitrary constant value (even a “dead neuron model” predicting a constant firing rate of 0 spikes/s) will have an *SPE* of zero and might on that basis be judged to perform better than other models generating noisy but fairly reasonable predictions (see Figure 1).

Of the three different types of error just discussed, large bias, poor scaling, small covariance, *SPE* is sensitive to two, covariance and scaling, although it is particularly excessively large, but not excessively small, scaling, that will drive *SPE* values down. Perhaps it is inevitable that single performance measures which are sensitive to multiple different types of error become very difficult to interpret as soon as performance becomes suboptimal. To an extent, whether one deems it preferable to have an error metric that is sensitive to bias, scaling and low covariance all at once, or whether one chooses a metric that is more specific in its sensitivity to only one of type of error is a matter of personal preference as well as of what one is hoping to achieve, but joint sensitivity to multiple different types of error is certainly problematic when the measure is to be used for model comparison, given that the relative weighting of the different types of error in the metric may not be readily apparent and it is unlikely to reflect how problematic the different types of error are in modeling. A constant bias, which would, for example, be heavily penalized by the *CD* metric discussed at the beginning of this section, can be easily fixed by adding or subtracting a constant value from the predictions. Similarly, scaling errors can be easily fixed by multiplication by a scalar. These two types of error pertain only to the relatively uninteresting stationary statistical properties of the data. They are in some sense trivial, and easily remedied through a simple linear adjustment. Low covariance, in contrast, is indicative of a much more profound inability of the model to capture the nature or dynamics of the neural stimulus-response relationships. In our opinion, the assessment of model performance should therefore rely first and foremost measures which are highly sensitive to poor covariance and insensitive to bias or scaling, and we discuss measures which have these properties in the next section. If needed, these could then be supplemented with additional metrics that can diagnose biases or scaling errors.

## 4. Absolute and normalized correlation coefficient

Another measure widely used in statistics, Pearson's product-moment correlation coefficient can also be used to assess the similarity of two time-varying signals. The correlation coefficient quantifies the linear correlation and maps it to a value between −1 and +1. To distinguish it from a normalized variant that will be used later in this section, the (absolute) correlation coefficient will from now on be abbreviated as *CC*_*abs*_. It is defined as:

(8) $$CC_{abs}=\frac{Cov(X,Y)}{\sqrt{Var(X)Var(Y)}}$$

*CC*_*abs*_ satisfies many of the criteria that one might desire in a good measure of model performance. It quantifies the similarity between observation and prediction, it is bounded between −1 and +1, and it can be interpreted easily and unambiguously. The normalization by the square root of the variances makes *CC*_*abs*_ insensitive to scaling errors, and the formulae for *Var*() and *Cov*() have subtractions of means built in that make *CC*_*abs*_ insensitive to bias, so that only the ability of *Y* to follow trends *X* is being quantified. However, like *VE*, it does not isolate model performance from prediction accuracy, which is inevitably limited by neural variability. In other words *CC*_*abs*_ might be small either because the model predictions *Y* are poor, or because the measured neural responses *X* are fundamentally so noisy that even an excellent model cannot be expected to achieve a large *CC*_*abs*_. This was also noted by Hsu and colleagues who went on to develop an approach to quantify and account for the inherent noise in neural data (Hsu et al., 2004). Specifically, they introduced a method for normalizing coherence and correlation to the neural variability, which has later been applied as a performance measure (Touryan et al., 2005; Gill et al., 2006). Hsu and colleagues define the normalized correlation coefficient as follows (Hsu et al., 2004)^2^:

(9) $$\begin{array}{l} CC_{norm}=\frac{CC_{abs}}{CC_{max}}\text{ with}\\ CC_{max}=\sqrt{\frac{2}{1+\sqrt{\frac{1}{CC_{half}^{2}}}}}\overset{CC_{half}>0}{=}\sqrt{\frac{2}{1+\frac{1}{CC_{half}}}} \end{array}$$

Where *CC*_*max*_ is the maximum correlation coefficient between the recorded firing rate *y* and the best prediction *ŷ* that a perfect model could theoretically achieve. More specifically, *CC*_*max*_ is the correlation coefficient between the recorded firing rate *y* (which is based on *N* trials) and the true (but unknown) underlying firing rate function, which could only be determined precisely if the system was completely stationary and an infinite number of trials could be conducted (*N* → ∞). Even though the true underlying firing rate function can therefore usually not be determined with high accuracy through experiments, useful estimates of *CC*_*max*_ can nevertheless be calculated using the formulae in Equation 9. Following the methods of Hsu et al. (2004), *CC*_*half*_ is determined by splitting the data set into halves, and calculating the correlation coefficient between the PSTH constructed from the first half and the PSTH constructed from the second half of the trials. This approach determines *CC*_*max*_ by effectively extrapolating from *N* trials to the value that would be expected for *N* → ∞.

Note that there are $\frac{1}{2}\left(\begin{array}{c} N\\ N/2 \end{array}\right)\;$ different ways to choose *N*∕2 out of *N* trials, and each such split of the data will yield a slightly different value for *CC*_*half*_. Thus, in theory, the best estimate would average over all possible values of *CC*_*half*_ calculated for each possible split. In practice, this resampling technique can be computationally expensive, given the fact that there are already 92, 378 combinations for *N* = 20 trials. Averaging over a smaller number of randomly chosen splits may often be sufficient, but this yields an imprecise estimation of *CC*_*max*_.

In summary, *CC*_*norm*_ provides a feasible method for capturing model performance independently of noise in the neural responses. It gives values bounded between -1 and +1 (in practice, they are bounded between 0 and +1, as model predictions are either correlated or not correlated, but typically not anti-correlated to the firing rate). Furthermore, the measure lends itself to unambiguous interpretation, and its limitations are well-known. Finally, it is normalized so that its value does not depend on the variability of a particular data set. Thus, the normalized correlation coefficient *CC*_*norm*_ fulfills the criteria for a useful measure of model performance, but its current definition is based in a laborious and potentially imprecise resampling technique.

## 5. A consolidated approach to quantifying neural variability

As will have become clear in the previous sections, the two measures *SPE* and *CC*_*norm*_ follow the same logic in that both measure prediction accuracy and normalize it by a quantification of the inherent reproducibility of the neural responses that are to be modeled (*SP* or *CC*_*max*_, respectively). In this section we will show that these two approaches of normalization not only follow the same logic, but are mathematically largely equivalent. This provides a deeper insight into the underlying concept and gives rise to a more elegant and efficient technique to normalize the correlation coefficient.

Following the methods of Sahani and Linden (2003)^3^, the signal power *SP* (i.e., the deterministic part of the recorded firing rate *y*) can be expressed as:

(10) $$\begin{array}{lll} SP & =\frac{1}{N-1}\left(N\times Var\left(y\right)-TP\right)\; & \; \end{array}$$

(11) $$\begin{array}{lll} & =\frac{1}{N-1}\left(N\times Var\left(\frac{1}{N}\overset{N}{\underset{n=1}{\sum }}R_{n}\right)-\frac{1}{N}\overset{N}{\underset{n=1}{\sum }}Var\left(R_{n}\right)\right)\; & \; \end{array}$$

(12) $$\begin{array}{lll} & =\frac{1}{N-1}\left(N\times \frac{1}{N^{2}}Var\left(\overset{N}{\underset{n=1}{\sum }}R_{n}\right)-\frac{1}{N}\overset{N}{\underset{n=1}{\sum }}Var\left(R_{n}\right)\right)\; & \; \end{array}$$

(13) $$\begin{array}{lll} & =\frac{1}{N-1}\left(\frac{1}{N}\times Var\left(\overset{N}{\underset{n=1}{\sum }}R_{n}\right)-\frac{1}{N}\overset{N}{\underset{n=1}{\sum }}Var\left(R_{n}\right)\right)\; & \; \end{array}$$

Where *TP* is the total power (i.e., the average variance of a single trial) and *R*_*n*_ is the recorded neural response of the *n*th trial. Since the normalization factor of *SPE* is the inverse of *SP* it will be convenient to express it as:

(14) $$\frac{1}{SP}=\frac{N(N-1)}{Var\left(\sum_{n\;=\;1}^{N}R_{n}\right)-\sum_{n\;=\;1}^{N}Var(R_{n})}$$

Furthermore, using Equation 14 the ratio of the noise power *NP* over *SP* can be expressed as:

(15) $$\begin{array}{ll} \frac{NP}{SP}=\frac{TP-SP}{SP}=\frac{TP}{SP}-1=\frac{\left(N-1\right)\times \overset{N}{\underset{n=1}{\sum }}Var\left(R_{n}\right)}{Var\left(\overset{N}{\underset{n=1}{\sum }}R_{n}\right)-\overset{N}{\underset{n=1}{\sum }}Var\left(R_{n}\right)}-1 & \; \end{array}$$

For *CC*_*norm*_ the normalization factor is the inverse of *CC*_*max*_ and, following the methods of Hsu et al. (2004), it is currently determined with an indirect resampling method using Equation 9. We will now show how *CC*_*max*_ can be computed directly by exploiting the relation between *SPE* and *CC*_*norm*_.

The coherence $\gamma_{AB}^{2}$ between a source signal *A* and a noisy recording *B* of this signal can be related to the signal-to-noise ratio, i.e., the coherence is just a function of the noise process itself (see Marmarelis, 1978 for details). In the context of neural recordings, Hsu et al. (2004) used this relation to express the coherence of the true (but unknown) underlying firing rate function (the source *A*) to the observed PSTH (the noisy recording *B*) as a function of the signal-to-noise ratio of the recording. They quantified this in terms of signal power of the frequency domain signals, but since the power of corresponding time and frequency domain signals is identical, we can rewrite their expression (see formulas 5 and 6 of Hsu et al., 2004) directly in terms of *NP* and *SP* to get:

(16) $$\gamma_{AB}^{2}=\frac{SP}{SP+\frac{1}{N}NP}$$

The derivation of the coherence function between the true underlying firing rate function and the observed neural response is analogous for the squared correlation coefficient between both signals (also see Hsu et al., 2004 for details on this analogy). Thus, we can apply the same principle to express the the inverse of *CC*_*max*_ as:

(17) $$\frac{1}{CC_{max}}=\sqrt{1+\frac{1}{N}\times \frac{NP}{SP}}$$

Combining Equation 17 with Equation 15 now allows us to express the inverse of *CC*_*max*_ as:

(18) $$\begin{array}{lll} \frac{1}{CC_{max}} & =\sqrt{1+\frac{1}{N}\left(\frac{\left(N-1\right)\times \overset{N}{\underset{n=1}{\sum }}Var\left(R_{n}\right)}{Var\left(\overset{N}{\underset{n=1}{\sum }}R_{n}\right)-\overset{N}{\underset{n=1}{\sum }}Var\left(R_{n}\right)}-1\right)}\; & \; \end{array}$$

(19) $$\begin{array}{lll} & =\sqrt{1-\frac{1}{N}+\frac{\left(1-\frac{1}{N}\right)\times \overset{N}{\underset{n=1}{\sum }}Var\left(R_{n}\right)}{Var\left(\overset{N}{\underset{n=1}{\sum }}R_{n}\right)-\overset{N}{\underset{n=1}{\sum }}Var\left(R_{n}\right)}}\; & \; \end{array}$$

Based on Equation 8 and 9 the normalized correlation coefficient *CC*_*norm*_ between the recorded firing rate *y* and the model prediction *ŷ* can now be expressed as:

(20) $$\begin{array}{ll} CC_{norm}=\frac{CC_{abs}}{CC_{max}}=\frac{Cov\left(y,ŷ\right)}{\sqrt{Var\left(y\right)Var\left(ŷ\right)}}\frac{1}{CC_{max}} & \; \end{array}$$

(21) $$\begin{array}{lll} & =\frac{Cov\left(y,ŷ\right)}{\sqrt{Var\left(y\right)Var\left(ŷ\right)}}\sqrt{1-\frac{1}{N}+\frac{\left(1-\frac{1}{N}\right)\times \overset{N}{\underset{n=1}{\sum }}Var\left(R_{n}\right)}{Var\left(\overset{N}{\underset{n=1}{\sum }}R_{n}\right)-\overset{N}{\underset{n=1}{\sum }}Var\left(R_{n}\right)}}\; & \; \end{array}$$

(22) $$\begin{array}{lll} & =\frac{Cov\left(y,ŷ\right)}{\sqrt{Var\left(y\right)Var\left(ŷ\right)}}\sqrt{1-\frac{1}{N}}\sqrt{1+\frac{\overset{N}{\underset{n=1}{\sum }}Var\left(R_{n}\right)}{Var\left(\overset{N}{\underset{n=1}{\sum }}R_{n}\right)-\overset{N}{\underset{n=1}{\sum }}Var\left(R_{n}\right)}}\; & \; \end{array}$$

(23) $$\begin{array}{ll} =\frac{Cov\left(y,ŷ\right)}{\sqrt{Var\left(ŷ\right)}}\frac{\sqrt{1-\frac{1}{N}}}{\sqrt{\frac{1}{N^{2}}Var\left(\overset{N}{\underset{n=1}{\sum }}R_{n}\right)}}\; & \;\\ \sqrt{1+\frac{\overset{N}{\underset{n=1}{\sum }}Var\left(R_{n}\right)}{Var\left(\overset{N}{\underset{n=1}{\sum }}R_{n}\right)-\overset{N}{\underset{n=1}{\sum }}Var\left(R_{n}\right)}} & \; \end{array}$$

(24) $$\begin{array}{ll} =\frac{Cov\left(y,ŷ\right)}{\sqrt{Var\left(ŷ\right)}}\sqrt{\frac{N\left(N-1\right)}{Var\left(\overset{N}{\underset{n=1}{\sum }}R_{n}\right)}}\; & \;\\ \sqrt{1+\frac{\overset{N}{\underset{n=1}{\sum }}Var\left(R_{n}\right)}{Var\left(\overset{N}{\underset{n=1}{\sum }}R_{n}\right)-\overset{N}{\underset{n=1}{\sum }}Var\left(R_{n}\right)}} & \; \end{array}$$

(25) $$\begin{array}{lll} & =\frac{Cov\left(y,ŷ\right)}{\sqrt{Var\left(ŷ\right)}}\sqrt{N\left(N-1\right)}\sqrt{\frac{1}{Var\left(\overset{N}{\underset{n=1}{\sum }}R_{n}\right)-\overset{N}{\underset{n=1}{\sum }}Var\left(R_{n}\right)}}\; & \; \end{array}$$

(26) $$\begin{array}{lll} & =\frac{Cov\left(y,ŷ\right)}{\sqrt{Var\left(ŷ\right)}}\sqrt{\frac{N\left(N-1\right)}{Var\left(\overset{N}{\underset{n=1}{\sum }}R_{n}\right)-\overset{N}{\underset{n=1}{\sum }}Var\left(R_{n}\right)}}\; & \; \end{array}$$

(27) $$\begin{array}{lll} & =\frac{Cov\left(y,ŷ\right)}{\sqrt{Var\left(ŷ\right)}}\sqrt{\frac{1}{SP}}\; & \; \end{array}$$

**Figure 2:**
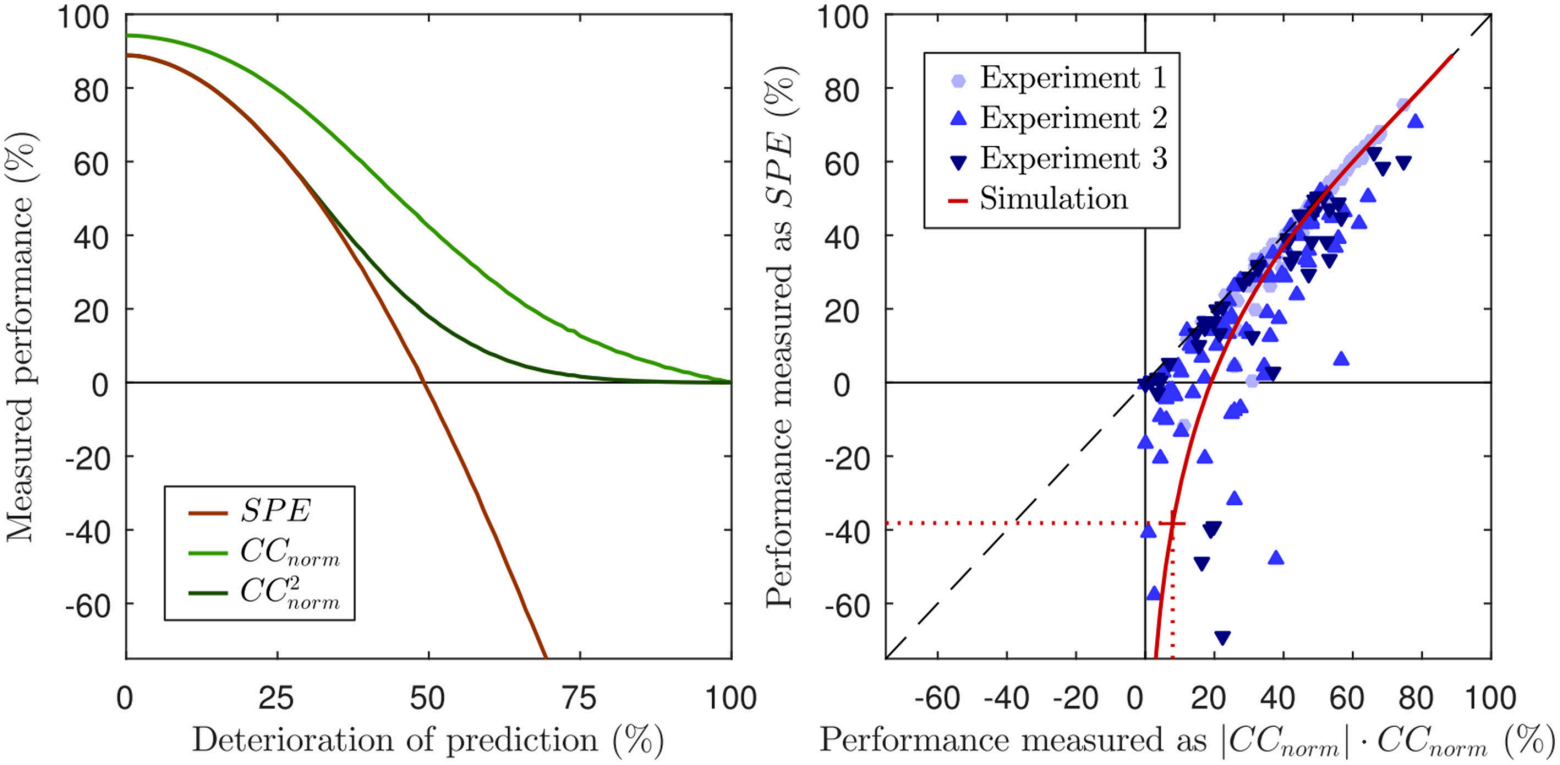
**Left panel: Same figure as the left panel of Figure 1, but including *CC*_*norm*_ and its squared values for reference**. Note that, for good predictions (values above ca 50%), $CC_{norm}^{2}$ and *SPE* are almost identical, both yielding very similar estimates of the proportion of “explainable variance explained.” This is as might be expected given the equality of Equation 7 and 28 when *ŷ* → *y*. However, as the prediction performance declines below 50%, $CC_{norm}^{2}$and *SPE* increasingly and sharply diverge. Right panel: Scatter plot of performance scores for predictions of neuronal responses from three different experiments. Each marker reflects the performance score of the prediction of the response of a single neuron. The black dashed line visualizes where *SPE* equals $CC_{norm}^{2}$. The values of *CC*_*norm*_ have been multiplied with their absolute value to demonstrate that negative values only occur for *SPE*, but not for *CC*_*norm*_. The solid red line shows the values for the simulation of Figure 1, the dotted red line and the red cross mark the performance scores of the 60% noise simulation of the lower right panel in Figure 1. Corresponding to the overlap of *SPE* and *CC*_*norm*_ for good predictions, the red line approaches the dashed black line.

In other words, we can now express *CC*_*norm*_ as a simple function of *SP*. The previous derivation also shows that both methods, *SPE* and *CC*_*norm*_, use the covariance to quantify the prediction accuracy and take the neural variability into account by normalizing with the signal power *SP*. This has several implications. First, *SPE* will not reveal more about the prediction accuracy than *CC*_*norm*_, because *SPE* and *CC*_*norm*_ quantify the similarity of the prediction and the neural response solely based on the covariance of both signals. It is well known that the (normalized) correlation coefficient is based on covariance, but it has hitherto not been made explicit that this is also the case for *SPE*. Note that *SPE* uses only the covariance to assess prediction accuracy and thus, cannot reveal more information about the similarity of both signals than *CC*_*norm*_. Second, how both measures quantify neural variability is not only related, but mathematically equivalent. Third, in order to calculate *CC*_*norm*_ it is not necessary to laboriously compute an approximation to *CC*_*max*_ from repeated subsampling of the data to generate computationally inefficient and potentially imprecise estimates of *CC*_*half*_. Instead, the normalization factor can be explicitly calculated with Equation 27, using Equation 13 for *SP* as suggested by Sahani and Linden (2003). The close relationship between both measures can also be visualized by squaring *CC*_*norm*_ (left panel of Figure 2).

In summary, *CC*_*norm*_ as defined in Equation 27 provides an insightful measure of model performance. It quantifies the prediction accuracy using the covariance and isolates model performance by taking the amount of intrinsic variability in the observed neural responses into account. It is in theory bounded between -1 and 1, and in practice values below zero are very rarely observed. If they do occur, their interpretation is unambiguous: negative *CC*_*norm*_ implies anticorrelation between prediction and data. *CC*_*norm*_ thus behaves uniformly well whether called upon to quantify the performance of good and of poor models, in contrast to *SPE* which behaves well, and very similarly to *CC*_*norm*_, for good models, but becomes increasingly harder to interpret as model performance declines.

## 6. Experimental validation

The previous sections show the problems caused by the missing lower bound of *SPE* from a theoretical point of view and illustrate them with a simulation (Figure 1). This section demonstrates the implications from a practical point of view by comparing the predictive performance of models for the activity of single neurons in the auditory system in three different experimental settings.

### 6.1. Neural recordings

All animal procedures were approved by the local ethical review committee and performed under license from the UK Home Office. Ten adult pigmented ferrets (seven female, three male; all >6 months of age) underwent electrophysiological recordings under anesthesia. Full details are as in the study by Bizley et al. (2010). Briefly, we induced general anesthesia with a single intramuscular dose of medetomidine (0.022 mg/kg/h) and ketamine (5 mg/kg/h), which was maintained with a continuous intravenous infusion of medetomidine and ketamine in saline. Oxygen was supplemented with a ventilator, and we monitored vital signs (body temperature, end-tidal CO2, and the electrocardiogram) throughout the experiment. The temporal muscles were retracted, a head holder was secured to the skull surface, and a craniotomy and a durotomy were made over the auditory cortex. We made extracellular recordings from neurons in primary auditory cortex (A1) and the anterior auditory field (AAF) using silicon probe electrodes (Neuronexus Technologies) with 16 or 32 sites (spaced at 50 or 150 μ*m*) on probes with one, two, or four shanks (spaced at 200 μ*m*). We clustered spikes off-line using klustakwik (Kadir et al., 2014); for subsequent manual sorting, we used either spikemonger (an in-house package) or klustaviewa (Kadir et al., 2014). The time-discrete neuronal firing rate was approximated by binning spikes in 5 ms windows and averaging the spike count in each bin over all trials (compare to Equation 1).

### 6.2. Acoustic stimuli

Natural sounds were presented via Panasonic RPHV27 earphones, which were coupled to otoscope specula that were inserted into each ear canal, and driven by Tucker-Davis Technologies System III hardware (48 kHz sample rate). The sounds had root mean square intensities in the range of 75–82 dB SPL. For Experiment 1, we presented 20 sound clips of 5 s duration each, separated by 0.25 s of silence. Sound clips consisted of animal vocalizations (ferrets and birds), environmental sounds (water and wind) and speech. The presentation of these stimuli was repeated in 20 trials. For Experiments 2 and 3, we presented 45 sound clips of 1 s duration, again separated by gaps of silence. The sound clips consisted of animal vocalizations (sheep and birds), environmental sounds (water and wind) and speech. The presentation of these stimuli was repeated in 10 trials. The silent gaps and the first 0.25 s thereafter have been removed from the data set.

### 6.3. Neuronal modeling

For Experiment 1, the responses of 119 single neurons were predicted with an LN model, a widely used class of models comprising a linear and a nonlinear stage (Chichilnisky, 2001; Simoncelli et al., 2004). The linear stage fits a spectro-temporal receptive field (STRF), which is a linear filter that links the neuronal response to the stimulus intensities of 31 log-spaced frequency channels (with center frequencies ranging from 1 to 32 kHz) along the preceding 20 time bins (covering a total of 100 ms stimulus history). The linear stage was fitted using GLMnet for Matlab (Qian et al.; see http://web.stanford.edu/~hastie/glmnet_matlab/). The nonlinear stage fits a sigmoidal nonlinearity to further maximize the goodness of fit to the neural response using minFunc by Mark Schmidt (University of British Columbia, British Columbia, Canada; http://www.di.ens.fr/~mschmidt/Software/minFunc.html). For Experiment 2, the same model class was used to predict the response of 77 single neurons. For Experiment 3, the responses of 43 single neurons were model with a standard neural network comprising 620 units in the input layer (31 frequency channels times 20 time bins of stimulus history), 20 hidden units and a single output unit. Hidden units and the output unit comprised a fixed sigmoidal nonlinearity. The connection weights of the network were fitted with backpropagation using the Sum-of-Functions Optimizer (Sohl-Dickstein et al., 2013). Both, the STRF weights of the LN models and the connection weights of the neural networks were regularized with a penalty term on the L2-norm in order to avoid overfitting. In all cases, models were trained and tested using a cross-validation procedure. All free model parameters were fitted on a training set comprising 90% of all data. The predictive performance of a model for a given neuron was assessed by measuring *SPE* and *CC*_*norm*_ for the model predictions of the neural response to the remaining 10% of the data set. This procedure was repeated 10 times, each time with a distinct 10% of data. The model performance was computed as the mean across all 10 performance measurements.

### 6.4. Results

We predicted neuronal responses to acoustic stimuli with different model classes in order to address the question how the choice of a performance measure affects the interpretability of the results in a practical setting. To this end, we measured the predictive performance of models with two different methods, *SPE* and *CC*_*norm*_. The right panel of Figure 2 shows a scatter plot in which each marker indicates the performance scores that the respective measures assign to a given prediction for a given neuron. Instead of raw *CC*_*norm*_ values, here we chose to plot the signed square of *CC*_*norm*_ as a percentage on the x-axis. This choice is motivated by the fact that the square of the correlation coefficient, also known as the coefficient of determination, quantifies the “proportion of variance explained” by a statistical regression model, and $CC_{norm}^{2}\times 100$ should thus be interpretable directly as a measure of “percent explainable variance explained” by the model. We plot the signed square to ensure that there are no artificial constraints keeping the *x*-values positive: the fact that there x-range of the data is entirely positive while the y-range extends well into negative territory veridically reflects the way the respective underlying metrics, *CC*_*norm*_ and *SPE*, behave in practice. For those cases in which the model predicts the actual neuronal response quite well, one can observe a very tight relation between the SPE value and the signed squared value of *CC*_*norm*_, i.e., both provide very similar, sensible measures of “percent explainable variance explained.” However, as expected from the theoretical analysis of both measures in the previous sections, this relation diminishes for cases in which the models poorly predicted the neuronal response. For those cases where there is little or no correspondence between the prediction and the response, the value of *CC*_*norm*_ approaches zero (by definition), and for some of those cases, the value of *SPE* also approaches zero, but for many others the *SPE* value becomes a large negative number. Substantially negative *SPEs* are seen even for some cases for which the |*CC*_*norm*_| × *CC*_*norm*_ indicates that the model was able to capture as much as 20–30% of the explainable, stimulus driven variability in the neural firing rate. Thirty percent variance explained may not be a stellar performance for a model, but it certainly does not seem deserving of a negative test score. Indeed, the experimental results are generally in accordance with the simulation in general, shown as a red line in the right panel of Figure 2. The simulation is identical to the one in Figure 1. To simulate *SPE* and *CC*_*norm*_ for a wide range of good and bad predictions, a good prediction was deteriorated by adding an increasing amount of white noise. Just as for the data from the three experiments, *SPE* values match the square of *CC*_*norm*_ for good predictions, but go deep into negative values for noisy predictions. For comparison, the *SPE* and *CC*_*norm*_ values of the example in the bottom right panel of Figure 1 (60% noise added) are marked with dotted lines in the right panel of Figure 2. In summary, the analysis of the experimental data from three experiments validate the theoretical analysis of the previous sections.

Figure 2 also visualizes the practical implications of the missing lower bound of *SPE*. *SPE* was from its inception described to be a “quantitative estimate of the fraction of stimulus-related response power captured by a given class of models” (Sahani and Linden, 2003). This interpretation is in conflict with values below zero because a fraction of a signal power cannot be negative. Furthermore, as was discussed in the previous sections, it is even difficult to assign an unambiguous interpretation to small or negative *SPE* values because a variety of poor models which vary widely in the size of their residual error can have similar small or negative *SPE*s, and may have *SPE*s below those of constant mean firing rate models of arbitrary value with an *SPE* of zero (including the “dead neuron model”), even if their residual error is smaller than that of these null models. If researchers are trying to quantify how well a particular class of models can describe the response properties of a sizeable sample population of neurons, a small number of somewhat spurious very negative values can heavily affect the overall population mean. For instance, the mean *SPE* value across the population of 77 neurons in Experiment 2 is just 15%, because a few very negative values drag down the average. But, as we have discussed in section 6, much of the negativity in those *SPE* values simply reflects a large variance in the predictions, which on its own is not very relevant, and constraining the *SPE* to values of zero or above would raise the mean performance by more than a quarter to over 19%.

## 7. Conclusion

Inter-trial variability of neural responses to repeated presentations of stimuli poses a problem for measuring the performance of predictive models. The neural variability inherently limits how similar one can expect the prediction of even a perfect model to be to the observed responses. Thus, when using prediction accuracy as a measure of performance, inherent response variability is a confound, and the need to control for this has been widely acknowledged (e.g., Panzeri and Treves, 1996; Sahani and Linden, 2003; Hsu et al., 2004; David and Gallant, 2005; Laudanski et al., 2012).

Different approaches for taking neural variability into account when measuring model performance have been developed. To get an unbiased estimate of *mutual information*, Panzeri and Treves (1996) have suggested a method to extrapolate information content to an infinite number of trials (also see Atencio et al., 2012). Sahani and Linden have developed the very insightful decomposition of the recorded signal into *signal power* and *noise power* (Sahani and Linden, 2003). This has lead to the *signal power explained (SPE)*, a measure based on *variance explained* which discounts “unexplainable” neural variability. This measure has been widely adopted, albeit under various names such as *predictive power, predicted response power, and relative prediction success* (Sahani and Linden, 2003; Machens et al., 2004; Ahrens et al., 2008; Asari and Zador, 2009; Rabinowitz et al., 2012). Also, it has been used as a basis for specific variants of measures for model performance (Haefner and Cumming, 2009). Focusing on coherence and the correlation, Hsu and colleagues have developed a method to normalize those measures by their upper bound (*CC*_*max*_), which is given by the inter-trial variability (Hsu et al., 2004). This yields the *normalized correlation coefficient* (*CC*_*norm*_). Following their suggestion, the upper bound can be approximated by looking at the similarity between one half of the trials and the other half of the trials (*CC*_*half*_). This measure has also been used by Gill et al. (2006) and Touryan et al. (2005). Others have used the absolute correlation coefficient and controlled for inter-trial variability by comparing the absolute values with *CC*_*half*_ (Laudanski et al., 2012).

In this study we have analyzed in detail two measures of model quality that account for neural response variability, *SPE* and *CC*_*norm*_. We have revealed the shortcomings of *SPE*, which has no lower bound and can yield undesirable negative values even for fairly reasonable model predictions. Furthermore, we have uncovered the close mathematical relationship between *SPE* and *CC*_*norm*_, consolidated both approaches and arrived at several insights. First, both measures quantify prediction accuracy using the covariance (and *only* using covariance). Second, both measures quantify neural variability using the *signal power* (*SP*) (and *only* using *SP*). Third, when the variance of the prediction error approaches zero, *SPE* becomes identical to the square of *CC*_*norm*_. And finally, it is not necessary to approximate *CC*_*max*_ using computationally expensive and inexact resampling methods because *CC*_*norm*_ can be calculated directly via *SP*:

(28) $$\begin{array}{lllll} CC_{abs}=\frac{Cov\left(y,ŷ\right)}{\sqrt{Var\left(ŷ\right)Var\left(y\right)}} & \; & CC_{norm}=\frac{Cov\left(y,ŷ\right)}{\sqrt{Var\left(ŷ\right)SP}} & \; & \; \end{array}$$

(29) $$\;SP=\frac{Var\left(\sum_{n=1}^{N}R_{n}\right)-\sum_{n=1}^{N}Var(R_{n})}{N(N-1)}$$

This consolidated definition of *CC*_*norm*_ is not only more elegant, precise, and efficient, but it also sheds light on how *CC*_*norm*_ can be interpreted. It is almost identical to the well-known Pearson's correlation coefficient *CC*_*abs*_, but the variance (power) of the recorded signal is replaced with the *signal power SP*, i.e., the deterministic and thus predictable part of the signal. As demonstrated, using *SPE* as a measure of model performance can yield misleading results and will limit interpretability of the results. However, *CC*_*norm*_ has been shown to fulfill the criteria of Section 2 for insightful measures: it is bounded, interpretable, and comparable across data sets. Thus, *CC*_*norm*_ is a well-defined and helpful tool to assess model performance^4^.

Note, however, that *CC*_*norm*_ cannot be estimated accurately if the data are excessively noisy. Equation 28 requires *SP* to be large enough to estimate with reasonable accuracy. For very noisy data or too few trials, observed *SP* values can become dominated by sampling noise, and may then behave as near zero random numbers. This would render *CC*_*norm*_ estimates unstable, allowing them to become spuriously large (if *SP* is small and underestimates the true value) or even imaginary (if the *SP* underestimate is severe enough to become negative). Thus, if *SP* or *CC*_*max*_ are small or have a very wide confidence interval, *CC*_*norm*_ values must be treated with caution.

## 8. Author contributions

OS: initiated the project; developed methodology; wrote and tested code implementing methods; analyzed method performance both analytically and through experiment; lead author on paper. NH, BW, AK, JS: guided research, co-wrote manuscript.

### Conflict of interest statement

The authors declare that the research was conducted in the absence of any commercial or financial relationships that could be construed as a potential conflict of interest.

## Footnotes

^1^Please note that we do not use the notation of Sahani and Linden (2003). However, all definitions are identical. Sahani and Linden define the *power P* of a signal *r* as the “average squared deviation from the mean: $P\left(r\right)=\langle {\left(r_{t}-\langle r_{t}\rangle \right)}^{2}\rangle$” where 〈.〉 denotes the mean over time. This is identical to the variance of the signal, which we use.

^2^The expression for *CC*_*max*_ can be derived from the work of Hsu et al. (2004) in two steps. First, Equations 6 and 8 from Hsu et al. (2004) are combined and solved for $\gamma_{A{\bar{R}}_{M}}$. Second, the analogy of the coherence γ^2^ and the squared correlation coefficient *CC*^2^ allows to replace $\gamma_{A{\bar{R}}_{M}}$ with *CC*_*max*_ and $\gamma_{{\bar{R}}_{1,M∕2}{\bar{R}}_{2,M∕2}}$ with *CC*_*half*_. In the notation of Hsu and colleagues $\gamma_{A{\bar{R}}_{M}}^{2}$ denotes the coherence of the mean response over *M* trials with the true (but unknown) underlying firing rate *A*, i.e., the maximum achievable coherence of a perfect model.

^3^Again, please note that Sahani and Linden (2003) use $\bar{r^{\left(n\right)}}$ to denote the average over trials. In order to facilitate the reformulation of the equation we do not use this abbreviated notation. Despite this difference in notation, this definition of *SP* is identical to the definition provided by Sahani and Linden (Equations 1 on Page 3).

^4^Matlab code for all measures can be found on GitHub: https://github.com/OSchoppe/CCnorm.
